# Psychological distress in women with recurrent spontaneous abortion: A case-control study

**DOI:** 10.4274/tjod.galenos.2019.88899

**Published:** 2019-10-10

**Authors:** Hajar Adib-Rad, Zahra Basirat, Mahbobeh Faramarzi, Amrollah Mostafazadeh, Ali Bijani

**Affiliations:** 1 Babol University of Medical Sciences, Institute of Health Research, Infertility and Reproductive Health Research Center, Babol, Iran; 2 Babol University of Medical Sciences, Institute of Health Research, Cellular and Molecular Biology Research Center, Babol, Iran; 3 Babol University of Medical Sciences, Institute of Health Research, Social Determinants of Health Research Center, Babol, Iran

**Keywords:** Recurrent spontaneous abortion, anxiety, depression, intolerance of uncertainty

## Abstract

**Objective:**

The aim of the present study was to evaluate psychological problems in women with recurrent spontaneous abortion (RSA).

**Materials and Methods:**

In this case-control study, 115 women with RSA were assigned to the case group and 240 non-pregnant women comprised the control group. The revised version of the Symptom Checklist-90 (SCL-90-R) and the Intolerance of Uncertainty scale (IUS) were used for assessing mental health problems.

**Results:**

The results showed that the mean Global Severity Index (GSI) of the SCL-90-R and the IUS scores in the case and control groups were 109.10±59.85 and 68.91±22.17, and 82.98±52.99 and 59.19±23.01, respectively. GSI was the strongest predictor of RSA [odds ratio (OR)=6.43; 95% confidence interval (CI): 3.52-11.72]. The chance estimate of RSA was approximately 2.1 times higher in women in rural areas (OR=2.07; 95% CI: 1.16-3.69), and 2 times higher at 12 months after the last pregnancy (OR=1.99; 95% CI: 1.42-2.78).

**Conclusion:**

Psychological problems are greater after RSA. Therefore, it is suggested that the treatment of RSA emphasizes psychological counseling and psychological management.

**PRECIS:** The loss of a desired pregnancy is a considerable negative life occurrence, and this problem may cause important physical and psychological distress.

## Introduction

Infertility and recurrent spontaneous abortion (RSA) are two challenging issues in the field of obstetrics and gynecology^(1,2)^. The perception of infertility has received great attention as a psychological problem^(3,4)^. It is considered as one of the numerous difficulties that patients should receive the best services in the diagnosis, treatment, and psychological support^(5)^. RSA is one of the most important problems related to infertility. It is defined as two or more consecutive pregnancy losses^(6)^. According to the American Society for Reproductive Medicine Practice Committee, RSA includes clinical abortion that is ascertained by ultrasound or histology^(7)^. RSA occurs due to genetic or uterine problems, thrombophilia, autoimmune endocrine diseases, infections, and several environmental factors^(8)^. Further, all cases of unknown infertility are often imputed to psychological causes^(9)^. The loss of a desired pregnancy is a considerable negative life occurrence, and this problem may cause notable physical and psychological distress^(10)^. Pregnancy loss is related with anxiety and distress, especially in women who experience RSA^(11)^. The prevalence of depression in abortion ranges from 15% to 33%^(12,13)^. In one study, researchers surveyed psychological adjustment to abortion and found that 50% of women with a history of abortion experienced depression and anxiety for several months^(14)^. Abortion may cause intolerance of uncertainty (IU) in women, which is a cognitive bias from a series of negative beliefs about uncertainty and its implications. In IU, a person perceives information in unclear circumstances and responds to it with a set of cognitive, emotional, and behavioral reactions^(15)^. Anxiety symptoms start immediately after abortion and continue until nearly 4-6 months later^(16)^. Additionally, while waiting for the next pregnancy, there is usually a high level of uncertainty and anxiety, which reduces the person’s ability to tackle problems^(17)^. According to the recommendations of the World Health Organization, women should wait for 6 months after an abortion and before attempting to become pregnant again^(18)^. However, about 50 to 80% of women become pregnant again soon after the abortion, and the next pregnancy is at risk of causing anxiety and depression^(19)^. Therefore, it is unclear if past RSA may be associated with depression or anxiety experienced by women. Thus, the future consequences of an RSA are unknown. Sham et al.^(20)^ reported no enhanced risk of psychiatric symptoms in subsequent abortion. Nevertheless, another study revealed that depression and anxiety after an abortion were significant predictors of symbolic anxiety and depression in the first trimester of the subsequent pregnancy^(21)^. Additionally, pregnancy loss may cause women to be concerned about the success of the next pregnancy^(22)^. Thus, owing to the impact of RSA, the diagnosis and management of anxiety and depression during the pregnancy after an abortion is as crucial as that of psychological distress during pregnancy^(23)^. Psychological support, also known as “tender loving care”, is considered essential for women who experience unexplained RSA^(24)^. Women without social support are at a higher risk of exhibiting psychological morbidity or symptoms after a pregnancy loss or infertility^(25,26)^. RSA is a distressing situation for infertile couples and frustrating for physicians. Accordingly, the European Society of Human Reproduction and Embryology and the Royal College of Obstetricians and Gynaecologists recommended offering supportive care during future pregnancies for women with unexplained RSA^(27)^. Many studies have been performed on depression in infertility but there are few studies on distress in RSA. Also, studies on the impact of psychological issues in RSA have reported conflicting findings. Therefore, the present study was conducted in Babol University of Medical Sciences in northern Iran to determine the impact of psychological problems on RSA.

## Materials and Methods

### Participants and procedure

This study was approved by the Research Ethics Committee of the Babol University of Medical Sciences (ID: MUBABOL.REC.2015.42). This case-control study was conducted from May 2015 to February 2017 in Babol, Iran. All patients signed the free and informed consent form. In total, 120 women with RSA were referred to the research center because of infertility. The women in the RSA group had primary infertility and had no children. RSA was defined as having two or more consecutive abortions in the first trimester of pregnancy. Out of those referred, 5 women were excluded owing to incomplete questionnaires, and the final case sample comprised 115 women. All women with known probable etiologies for RSA and known mental illnesses were excluded from the study. The inclusion criteria for the patients with RSA included having experienced at least two consecutive idiopathic abortions of a desired pregnancy with a sexual partner; regular menstruation; no history of polycystic ovary syndrome; normal gynecologic status; anatomy, and karyotype; normal levels of the anti-phospholipid antibody, anti-nuclear antibody, anti-cardiolipin antibody, anti-thrombin 3, lupus anti-coagulant, homocysteine, protein S, protein C, factor V Leiden, anti-thyroid peroxidase, thyroid hormones, and prolactin; and normal spermogram and karyotype of the sexual partner. Women without RSA who were referred to primary healthcare centers were selected as control subjects. These 265 healthy, non-pregnant women with at least one living child had no history of infertility, previous abortion, preterm deliveries, or stillbirths. Among them, 25 women were excluded from the study due to failure to complete the questionnaire, and a final sample of 240 women was used as the control group. The case and control groups were evaluated from three months to one year after abortion or childbirth, respectively.

### Demographics and questionnaires

All participants completed the sociodemographic information questionnaire, the revised version of the Symptom Checklist-90 (SCL-90-R), and the Intolerance of Uncertainty scale (IUS). For both groups, we collected information on the couple’s age, level of education, body mass index (BMI), occupation, residence, home ownership status, satisfaction with income, and gravidity and time passed since the last pregnancy. The SCL-90-R is one of the most widely used symptom questionnaires in the field of psychiatry. This self-reported questionnaire evaluates the following 9 symptoms: somatization, sensitivity, obsessive-compulsive disorder, aggression, phobic anxiety, paranoia, depression, anxiety, and psychotic tendency. The total score is evaluated as the Global Severity İndex (GSI). This checklist comprises 90 questions with 5 response options (0=not at all, 1=a little bit, 2=moderately, 3=quite a bit, 4=extremely)^(28,29)^. The IUS assesses cognitive bias that changes perceptions, interpretations, and individual reactions in uncertain situations based on the levels of cognitive, emotional, and behavioral responses in such situations. IU is defined as the predisposition to react negatively to an uncertain event or situation, independent of its probability of occurrence and of its associated consequences. IU reflects beliefs about the necessity of being certain, the capacity to cope with unpredictable change, and about adequate functioning in situations that are inherently ambiguous^(30)^. IUS is correlated with psychological problems such as anxiety and depression, but infertility and abortion, especially recurrent abortion, is a specific situation with high frequency of uncertainty events. Infertility is uncertainty as a diagnosis, uncertainty as successful treatments and uncertainty as future outcomes. Also, recurrent abortion is full of uncertainty conditions, such as uncertainty as to the cause and if related with disease, uncertainty regarding future pregnancy and future outcomes. The IUS is a 27-item questionnaire that assesses incompatible beliefs that lead to IU . Responses to items are made using a 5 point Likert scale (not at all, somewhat, medium, high, very high). The internal consistency of the scale was α=0.91, and its reliability was 78% in a previous study^(15)^. This scale classifies the nature of worry experienced by healthy into the following four categories: bearing down on ambiguous situations, positive beliefs about worry, cognitive avoidance, and negative problem orientation^(31)^.

### Statistical Analysis

Data analyses were performed using the Statistical Package for the Social Sciences (SPSS) 22.0 software package. The differences in sociodemographic characteristics and psychological distress between women with and without RSA were determined using the t-test and chi-square test. Analysis of variance (ANOVA) and the Tukey’s test were used to examine differences in psychological distress at 1-6, 7-12, and >12 months after abortion and delivery. Additionally, the predictive factors of RSA (age, GSI, education, time passed since last pregnancy, occupation, residence, and income) were examined using multiple logistic regression analysis. Also, Pearson correlation analysis was used to identify the significant relationship of IUS and SCL-90-R. A p value of less than 0.05 was considered significant.

## Results

The sociodemographic characteristics of the participants are presented in Table 1. There was no significant difference between the case and control groups regarding BMI, occupation, level of education, husband’s age and level of education, satisfaction with income, and home ownership status (Table 1). The mean global GSI on the SCL-90-R score in women with and without RSA were 109.10±59.85, and 82.98±52.99, respectively (p=0.0001). Also, the mean IUS score in women with and without RSA was 68.91±22.17, and 59.19±23.01, respectively (p=0.0001). There was a significant difference in location between the two groups. Therefore, we examined if their psychological distress varied based on location. Table 2 shows the mean and standard deviation on all subscales of the SCL-90-R and the IUS for the recognition of prior learning and control groups. It was observed that in the case group, the scores were higher in rural populations as compared with those in urban populations. In contrast, in the control group, the scores in rural populations were lower than those in urban populations (p=0.0001). ANOVA and Tukey’s test were used to assess differences in psychological distress at 1-6, 7-12, and >12 months after pregnancy loss and birth. In the group without RSA (control group), scores on all psychological distress subscales reduced significantly between 1-6 months to >12 months after birth (p=0.0001). However, in the RSA group, mental health problems remained stable even after 12 months since abortion. The Tukey’s test results for the different time periods since the last pregnancy are summarized in Table 3. Table 4 shows the predictive factors of RSA based on the findings of multiple logistic regression analysis in the first stage. Seven factors including age, GSI, education, time passed since last pregnancy, occupation, residence, and satisfaction with income were included in the analysis. The odds ratio (OR) of the GSI was good for RSA before the adjustment [OR=3.48; 95% confidence interval (CI)=(2.10-5.77), p<0.001] and this association was strengthened by considering other factors. The adjusted ORs for factors that were significantly associated with the chance of RSA are summarized in Table 4. Using this method, we excluded the variable of having age over 30 years in the second stage, jobs in the third stage, and satisfaction with income in the fourth stage, and only GSI [OR=6.43; 95% CI=(3.52-11.72)], time passed since last pregnancy (OR=1.99; 95% CI=1.42-2.78), education (OR=0.49; 95% CI=0.50-1.84), and residence (OR=2.07; 95% CI=1.16-3.69) remained in the model. After adjusting for other variables, the multiple logistic regression analysis showed that the chance of RSA was higher in women living in rural areas as compared with those living in urban areas (p=0.013), and in people with an educational level of high school diploma or less as compared with their counterparts (p=0.026). Also, Pearson correlation analysis showed a significant relationship of IUS with SCL-90-R (r=0.650, p<0.001).

## Discussion

Recurrent abortion and the postpartum period are serious time points for women^(32,33)^. In this study, we found that the incidence of psychological disorders was higher in women with recurrent abortions, as evident from their higher scores on the SCL-90-R and IUS as compared with those of the control group. These results are in agreement with those of the Kolte et al.^(34)^ who revealed that 8.6% of women with RSA, versus 2.2% of healthy women had moderate or severe depression. In another study that examined the psychological adjustment in couples with a history of recurrent miscarriage, the results showed that, according to Beck Depression index scores, women presented significantly higher levels of anxiety and depression as compared with men. Further, 25% of the women, versus 3.9% of the men, exhibited a high level of state anxiety as assessed using the State-Trait Anxiety Inventory (STAI-S ≥ 55), and 23.7% of the women, versus 5.3% of the men, exhibited a high level of trait anxiety (STAI-T ≥55)^(35)^. In Sugiura-Ogasawara et al.’s^(29)^ study, 305 women with a history of recurrent abortion first completed a set of questionnaires including the K6 (a new screening instrument for anxiety and mood disorders) and the SCL-90-R. Subsequently, 170 women received a description about a successful live birth, after which they answered the questionnaires again. The results showed that, in the first survey, 15.4% of the women exhibited anxiety or depression. Additionally, high scores on the K6 were correlated with high scores on all the subscales of the SCL-90-R. Further, in the 170 women who received a description of a successful live birth, all scores in the second survey were significantly lower as compared with the first survey, indicating an improvement in their depression levels^(29)^. In another study, results showed that a high score on the IUS were associated with an increased risk of opposing behaviors^(36)^. In addition, Carleton et al.^(37) ^reported that IUS scores in women with social anxiety disorder were higher than those in women with panic disorder (p<0.01). Furthermore, in one study on 151 members with primary social anxiety disorder, their IUS scores were evaluated before and after 12 weeks of cognitive behavioral therapy. The findings showed significantly lower scores after treatment^(38)^. These findings indicate that anxiety is higher in infertile women. Infertility and recurrent abortion can lead to a substantial amount of pressure on women. These women do not recover spontaneously, and they need appropriate diagnostic services, care, and psychological intervention. In our study, the control group exhibited a significant reduction in their scores on all subscales in psychology from 1-6 months to >12 months after birth; however, the same women with RSA remained stable even 12 months after abortion. Broen et al.^(39)^ showed that women who experienced a spontaneous abortion exhibited more distress between 10 days and 6 months after the miscarriage. Kagami et al.^(35)^ reported that depression increased from 8.9% in the ≤3 month period after miscarriage, and to 9.1% in the >3 month period; whereas, anxiety decreased after 3 months. In contrast to our study, Kolte et al.^(34)^ stated that, in 44.4% of women whose last pregnancy loss was six months ago, the time since abortion was not related to psychological factors. In another study, the effect of duration since last recurrent abortion was not evaluated with regard to mental health^(12)^. The above results indicate that abortion and mental health problems resulting from RSA may sustain even after one year. Therefore, psychological counseling and intervention are necessary for patients with RSA. In our study, the predictors of RSA included GSI, time passed since last pregnancy, education, and place of residence. Similarly, in a previous study, researchers reported that depression rates increased after adjusting for the level of education, income, age, and number of pregnancies [unadjusted OR=4.19, 95% CI=(2.52-6.98), adjusted OR: 5.53, 95% CI=(2.09-14.61)]^(34)^. How can we explain the higher distress in women living in rural areas? Rural and urban areas exhibit differences in terms of social culture. For instance, in rural areas, infertility is considered a stigma. Therefore, rural couples without children may feel more pressurized about being childless than those who live in cites. Supportive care from healthcare professionals can be effective in avoiding distress after pregnancy loss and during a new pregnancy^(40)^. One of the strengths of our study was the assessment of psychological problems in the two groups at 12 months and more after an abortion and normal delivery. In this period, psychological problems persisted in those with RSA, which provided proof of the need for social support and psychological counseling in this group. The other strong point of our study was the evaluation of psychological distress using the IUS scale, which has never been used in any study in this field.

### Study Limitation

The limitation of our study is that the case sample only included women who had experienced RSA in the first trimester due to the absence of cases of abortion in the second trimester. Therefore, it is suggested that future studies include psychiatric evaluations of women who experience abortion in the second trimester.

## Conclusion

In conclusion, the findings of our study showed that the psychological distress in women with RSA was higher after abortion, it persisted even after one year since the abortion, and it was of greater intensity in women from rural areas. Therefore, it is suggested that women with RSA be provided with psychological counseling to handle the distress they experience. Thus, the psychological management of distress in women with miscarriage must be included in the treatment of RSA.

## Figures and Tables

**Table 1:**
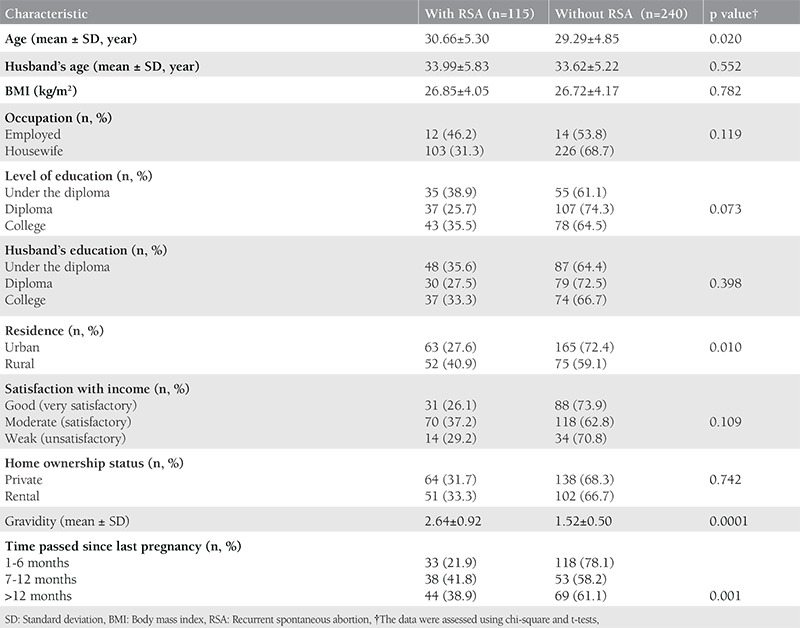
Sociodemographic characteristics in population study

**Table 2:**
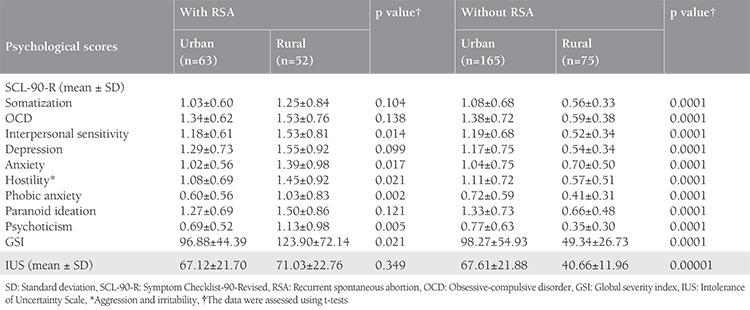
Psychological distress according to the residence in two groups

**Table 3:**
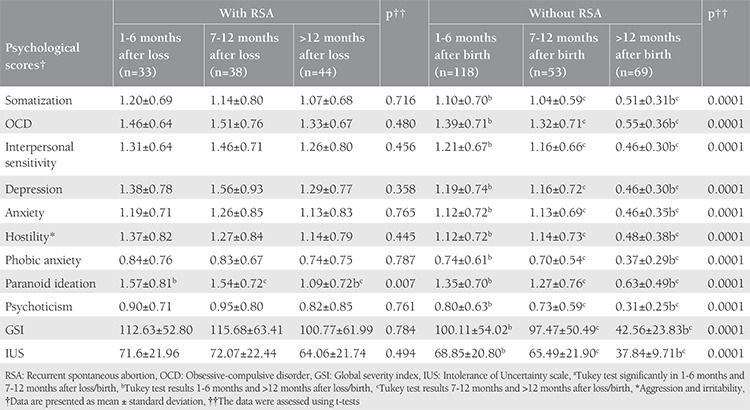
Trend of psychological distress after pregnancy loss/birth

**Table 4:**
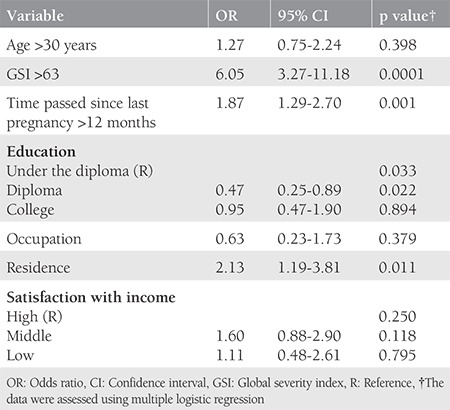
Predictive factors of recurrent spontaneous abortion in the multiple logistic regression analysis in the first stage
